# Diversity of Hybrid- and Hetero-Pathogenic *Escherichia coli* and Their Potential Implication in More Severe Diseases

**DOI:** 10.3389/fcimb.2020.00339

**Published:** 2020-07-15

**Authors:** Ana Carolina de Mello Santos, Fernanda Fernandes Santos, Rosa Maria Silva, Tânia Aparecida Tardelli Gomes

**Affiliations:** Disciplina de Microbiologia, Departamento de Microbiologia, Imunologia e Parasitologia, Escola Paulista de Medicina, Universidade Federal de São Paulo, São Paulo, Brazil

**Keywords:** *Escherichia coli*, intestinal infection, extraintestinal infection, hybrid, hetero-pathogenic, pathotypes, ExPEC, DEC

## Abstract

Although extraintestinal pathogenic *Escherichia coli* (ExPEC) are designated by their isolation site and grouped based on the type of host and the disease they cause, most diarrheagenic *E. coli* (DEC) are subdivided into several pathotypes based on the presence of specific virulence traits directly related to disease development. This scenario of a well-categorized *E. coli* collapsed after the German outbreak of 2011, caused by one strain bearing the virulence factors of two different DEC pathotypes (enteroaggregative *E. coli* and Shiga toxin-producing *E. coli*). Since the outbreak, many studies have shown that this phenomenon is more frequent than previously realized. Therefore, the terms hybrid- and hetero-pathogenic *E. coli* have been coined to describe new combinations of virulence factors among the classic *E. coli* pathotypes. In this review, we provide an overview of these classifications and highlight the *E. coli* genomic plasticity that results in some mixed *E. coli* pathotypes displaying novel pathogenic strategies, which lead to a new symptomatology related to *E. coli* diseases. In addition, as the capacity for genome interrogation has grown in the last few years, it is clear that genes encoding some virulence factors, such as Shiga toxin, are found among different *E. coli* pathotypes to which they have not traditionally been associated, perhaps foreshowing their emergence in new and severe outbreaks caused by such hybrid strains. Therefore, further studies regarding hetero-pathogenic and hybrid-pathogenic *E. coli* isolates are necessary to better understand and control the spread of these pathogens.

## Introduction

*Escherichia coli* is a gram-negative, facultative anaerobic rod, which produces catalase but not oxidase. Taxonomically, it belongs to class *Gammaproteobacteria*, order *Enterobacteriales*, and family *Enterobacteriaceae* (Adeolu et al., 2016). Bacteria of this species inhabit the intestinal tract of humans and other animals as an important member of their microbiota (Leimbach et al., 2013). Moreover, the high adaptive capacity of *E. coli* permits it to survive for long periods of no growth and in a variety of niches such as soil, water, food, and sediments (Leimbach et al., 2013). Although most are innocuous, some strains of this species are pathogenic and can cause intestinal or extraintestinal diseases, which are related to a variety of virulence genes acquired by the horizontal transfer of plasmids, pathogenicity islands, transposons, and bacteriophages (Kaper et al., 2004; Croxen and Finlay, 2010; Leimbach et al., 2013; Johnson and Russo, 2018).

The pathogenic *E. coli* strains are classified according to the infection site of isolation. Strains capable of causing diseases in the human intestinal tract are designated as diarrheagenic *E. coli* (DEC), which is subclassified into seven different pathotypes: enterotoxigenic *E. coli* (ETEC), enteroinvasive *E. coli* (EIEC), enteroaggregative *E. coli* (EAEC), and enteropathogenic *E. coli* (EPEC), both sub-grouped in typical and atypical Shiga toxin-producing *E. coli* (STEC), diffusely adherent *E. coli* (DAEC), and adherent-invasive *E. coli* (AIEC) (Kaper et al., 2004; Croxen et al., 2013; Leimbach et al., 2013; Gomes et al., 2016). Except for AIEC and DAEC, the differences among most of these pathotypes are typically due to specific virulence encoding genes that are directly related to the development of the disease and host symptomatology and are used for diagnostic purposes (Table 1). AIEC has been postulated as a cause of inflammatory bowel disease. However, at present, there is no consensus on this issue (Palmela et al., 2018; Perna et al., 2020), and the putative virulence factors described as involved in AIEC pathogenesis are common to strains isolated from extraintestinal infections (Martinez-Medina et al., 2009; Yang et al., 2017). Furthermore, although DAEC is a recognized enteric pathogen and the presence of genes encoding afimbrial adhesins are occasionally used for screening this pathotype, these genes are also present in other intestinal and extraintestinal pathogenic *E. coli* as well as in commensal strains, limiting their usefulness in defining the DAEC pathotype (Croxen et al., 2013).

**Table 1:** *Escherichia coli* pathotypes and the virulence genetic markers used for their classification^a^.

**Pathotype**	**Subgroup**	**Virulence genetic markers (function or virulence factor)**	**Observations**
EAEC	Typical (tEAEC)	*aggR* (transcriptional activator)	The occurrence of the aggregative adhesion pattern in HeLa/Hep-2 cells is the standard method to characterize EAEC
	Atypical (aEAEC)	*aatA* and *aaiG* (plasmid and chromosomal secretion system, respectively)	
EPEC	Typical (tEPEC)	*bfp* (bundle-forming pilus), *eae* (intimin), and absence of *stx* (Shiga toxin)	Characterized by the presence of LEE
	Atypical (aEPEC)	*eae* (intimin) and the absence of *bfp* and *stx*	
STEC	–	*stx* (Shiga toxin)	–
			The presence of LEE pathogenicity island confers to EHEC the ability to cause A/E lesion in the intestinal epithelium, as EPEC
	EHEC	s*tx* (Shiga toxin) and *eae* (intimin)	
ETEC	–	*elt* (heat-label toxin—LT) and/or *est* (heat-stable toxin—ST)	–
EIEC	–	*ipaH* (multicopy family of effectors present in invasion plasmid and chromosome)	–
DAEC^b^	–	*dra* or *afa* (AFA-Dr adhesins)	No virulence factor confirmed as determinant of diarrhea was described
AIEC^b^	–	Unknown	No virulence factor confirmed as determinant of inflammatory disease
ExPEC^c^	–	Presence of at least two of five genes: *pap* (P fimbriae), *sfa* (S fimbriae), *afa/dra* (AFA-Dr adhesins), *iuc/iut* (aerobactin), and *kpsMT*II (capsular group II)	Intrinsic virulent strains are lethal in an animal model of sepsis; not all extraintestinal isolates harbor this set of virulence genes
UPEC^d^	–	Simultaneous presence of the following genes: *yfcV* (fimbriae Yfc), *vat* (vacuolating autotransporter toxin), *chuA* (heme receptor), and *fyuA* (yersiniabactin)	Strains harboring these four genes can cause urinary tract infection in animal models; not all strains isolated from UTI harbor these four genes

a *The table displays the virulence factors often used in surveillance studies and the diagnosis of intestinal infections. It does not represent the complete list of virulence factors that each pathotype can harbor*.

b *The classification of DAEC and AIEC was not based on the presence of specific virulence factors; consequently, it is not possible to identify the hybrid/hetero-pathogenic strains of these pathotypes*.

c *All E. coli strains isolated from any extraintestinal infection are ExPEC. This term can also be used to classify strains isolated from diverse sources that harbor specific virulence factors and are intrinsically virulent, being lethal to mice in the animal model (Picard et al., 1999; Russo and Johnson, 2000; Johnson et al., 2003)*.

d *UPEC is the pathotype designation used to refer to ExPEC strains that were isolated from urinary tract infections or strains isolated from diverse sources that are capable of causing urinary tract infections in an animal model (Spurbeck et al., 2012; Johnson and Russo, 2018)*.

Unlike DEC, extraintestinal pathogenic *E. coli* (ExPEC) are defined primarily by their site of isolation. The most clinically important ExPEC groups are uropathogenic *E. coli* (UPEC), neonatal meningitis-associated *E. coli* (NMEC), avian pathogenic *E. coli* (APEC), and septicemic *E. coli* (SEPEC) (Ewers et al., 2007; Santos et al., 2013; Johnson and Russo, 2018). ExPEC strains can cause infection in diverse extraintestinal sites. Furthermore, a strain that causes urinary tract infection in humans can also cause infections in other human body sites or in animals, which makes the use of the term ExPEC more appropriate than the pathotype designation (Russo and Johnson, 2000). There is no single or set of virulence factors exclusively associated with a specific host or disease, but Picard et al. (1999) and Johnson et al. (2003) have shown that the ability of ExPEC strains to cause disease in immunocompetent subjects was associated with the presence of two among five virulence markers (Table 1). The strains that bear these traits were referred to as “intrinsic virulent” because they are considered more pathogenic than those that do not harbor these factors. Similarly, Spurbeck et al. (2012) have proposed a set of four other genes to identify ExPEC strains with uropathogenic potential (Table 1). In general, ExPEC virulence factors appear partially redundant, involved in the ability of these strains to colonize, evade immune system clearance, and survive in diverse extraintestinal sites (Croxen and Finlay, 2010; Leimbach et al., 2013; Johnson and Russo, 2018).

After the increased access to genome sequencing technologies, several pathogenic *E. coli* genomes became available. These data highlighted *E. coli* genomic plasticity and showed the distribution of virulence factors among the pathotypes, including those traits related to DEC definition. A remarkable example of plasticity was the *E. coli* strain involved in 2011's outbreak that displayed the characteristics of two different pathotypes and led to severe host symptoms. Consequently, the terms “hybrid” and “hetero-pathogens” have emerged to designate potentially more virulent strains that present a combination of virulence factors, which were previously believed to be specific to each *E. coli* pathotype. In this review, we bring together the reports on hybrid- and hetero-pathogenic *E. coli* strains and discuss their potential implication in more severe diseases.

## Defining Hetero-Pathogens and Hybrid-Pathogens Without Deconstructing Primary Concepts

Here we adopt the terms “hetero-pathogenic” or “hetero-pathogen” to refer to strains that harbor virulence genes that are characteristic of two or more DEC pathotypes. Hence, the hetero-pathogens are strictly entero-pathogens, and their designation is based on the presence of specific virulence factors-associated DEC pathotypes. Their definition is straightforward because genes that delineate DEC are well-defined. A limitation is that the absence of defined virulence markers prevents the inclusion of DAEC and AIEC in these definitions.

In contrast, “hybrid-pathogenic” or “hybrid-pathogen” strains exhibit both DEC and ExPEC defining virulence factors or, alternatively, are isolated from an extraintestinal infection and encode DEC defining virulence factors. The alternate criterion for designation as a hybrid-pathogen is required due to ambiguity in the gene sets required to define ExPEC strains.

## Combinations of Virulence Factors That Lead to More Severe Diseases

The current challenge concerning the hybrid- or hetero-pathogenic *E. coli* strain classification is understanding whether these virulence factors are, in fact, involved in disease development and have clinical relevance that could be considered in diagnosis. We present below some examples of virulence marker combinations that were associated with more severe diseases.

### EAEC/STEC

The higher virulence of the hetero-pathogenic EAEC/STEC group was highlighted when it caused the foodborne outbreak that started in Germany in 2011 and spread out to Europe and North America (Bielaszewska et al., 2011; Mellmann et al., 2011; Rasko et al., 2011), affecting more than 4,000 persons in 16 countries (Center for Disease Control Prevention, 2013). In Germany, as many as 3,816 cases were reported, 845 of which progressed to hemolytic uremic syndrome (HUS) and with 54 deaths (Frank et al., 2011).

Studies conducted in Germany and France showed no evidence of zoonotic origin for this outbreak and that this hetero-pathogen became well-established in the human population (Monecke et al., 2011; Wieler et al., 2011; Auvray et al., 2012). The success of this hetero-pathogen can be explained by its genetic background, which combines the EAEC O104:H4 and the STEC virulence profiles, comprising the aggregative adherence (AA) pattern and the Stx2 production (Bielaszewska et al., 2011; Mellmann et al., 2011; Rasko et al., 2011). Another factor that makes the EAEC/STEC outbreak alarming is that infections caused by this hetero-pathogen frequently progress to HUS, possibly because they present a set of proteins involved in intestinal colonization such as IrgA homologue adhesin (Iha), aggregative adherence fimbriae I (AAF/I), long polar fimbriae (Lpf), and different serine-protease autotransporters of Enterobacteriaceae (like Pic, SepA, etc.). These proteins could work synergistically to make these strains better colonizers, leading to persistent diarrhea and facilitating Shiga-toxin (Stx) absorption (Navarro-Garcia, 2014).

Besides that, a study conducted at the German National Reference Center with 2,400 STEC strains, isolated between 2008 and 2012, identified two additional hetero-pathogenic EAEC/STEC strains that were isolated from a patient with diarrhea and exhibited the AA pattern and the *stx* gene. One of these strains produced AAF/ IV, while a new type of aggregative fimbriae was described in the other (Prager et al., 2014; Lang et al., 2018). Furthermore, before the German outbreak, other EAEC/STEC hetero-pathogens were described in HUS cases associated with outbreaks that occurred in France (serotype O111: H2) and HUS and bloody diarrhea in Japan (serotype O89: HNM) (Boudailliez et al., 1997; Morabito et al., 1998; Iyoda et al., 2000).

### EHEC as a Long-Standing EPEC/STEC Hetero-Pathogen

The enterohemorrhagic *E. coli* (EHEC) pathotype is involved worldwide in many outbreaks, severe symptomatology, and lethal outcomes to the human host (Levine, 1987; Kaper et al., 2004; Croxen et al., 2013; Gomes et al., 2016). This pathotype has been classified based on different concepts. Some researchers classify any STEC strain as EHEC based only on the patients' clinical manifestations, including hemorrhagic colitis or HUS due to Stx production (Doughty et al., 2002; Navarro-Garcia, 2014; Krause et al., 2018; Lang et al., 2018; Torres et al., 2018). Others, instead, use molecular criteria based on the simultaneous presence of Stx and the locus of enterocyte effacement (LEE) pathogenicity island (PAI) to classify as EHEC (Kaper et al., 2004; Croxen et al., 2013; Sadiq et al., 2014; Silva et al., 2019).

Herein the EHEC nomenclature has been used to refer to the STEC subset that simultaneously harbors Stx and LEE. As STEC strains can cause diarrhea and HUS in human subjects independently of the presence of LEE (Luck et al., 2005; Croxen et al., 2013; Krause et al., 2018), this PAI is an accessory set of virulence genes that enhances STEC pathogenicity; thus, we can consider it as a hetero-pathogen. Nevertheless, LEE PAI is the major factor associated with the occurrence of attaching and effacing (A/E) lesion and diarrhea by EPEC strains (Croxen et al., 2013; Silva et al., 2019). LEE bears several virulence genes encoding, e.g., intimin (*eae*), a type 3 secretion system, and other effectors involved in A/E lesion formation, which are used to clinically classify pathogenic *E. coli* strains as EPEC (Table 1).

The EPEC pathotype is sub-grouped based on the presence of the bundle-forming pilus (BFP) in the strains classified as typical and, on its absence, in the strains classified as atypical. In general, EPEC/STEC hetero-pathogens comprise atypical EPEC carrying Stx (Croxen et al., 2013; Eichhorn et al., 2015; Gomes et al., 2016; Silva et al., 2019). The first description of a typical EPEC strain bearing Stx was reported by Gioia-Di Chiacchio et al. (2018), who identified eight *E. coli* strains isolated from birds that carried Stx2, LEE, and BFP simultaneously. The production of Stx2 was shown in all strains, and two strains induced A/E lesions in cell cultures. All these isolates belonged to the same serotype (O137:H6) and sequence type (ST2678).

### EPEC/ETEC

The first reported case of these hetero-pathogenic isolates was of a 6-month-old child presenting acute watery and bloody diarrhea (Dutta et al., 2015), a more severe symptom. The virulence of this strain was assessed *in vitro*, showing that it induced A/E lesions and produced a functional heat-label toxin (LT) (Dutta et al., 2015). In 2016, a new type of EPEC/ETEC strain bearing the heat-stable toxin (ST) instead of LT-encoding genes was reported in healthy cattle (Askari Badouei et al., 2016), but the ST expression was not evaluated. Hazen et al. (2017) reported the identification of four EPEC/ETEC hetero-pathogenic strains isolated from children, two of them being with diarrhea, one asymptomatic, and another with lethal outcome. The EPEC/ETEC strain isolated from the stools of the latter child presented the autotransporter *eatA* accessory gene of ETEC, which prompted the authors to suggest its consideration as a hetero-pathogen (Hazen et al., 2017). However, from our point of view, the presence of *eatA* is not an adequate criterion since this factor is neither an ETEC pathotype defining marker nor a marker related to the ETEC infection symptomatology. Although this is the most recent report concerning these pathogens, the study comprised strains that were isolated between 2008 and 2009 in Africa and Asia (Kotloff et al., 2012). Additionally, in all cases, the EPEC strains had acquired plasmids bearing the ETEC toxin-encoding genes.

### ExPEC/STEC

The ExPEC/STEC hybrid is a high-risk pathogen for humans due to the possibility of a systemic infection occurring in concomitance with HUS, which might be aggravated by the presence of a multidrug resistance phenotype, making treatment even more difficult. Reports on the occurrence of HUS just after *E. coli* urinary tract infection (UTI) are rare but have been noted in different countries since 1979 (reviewed by Lavrek et al., 2018). Many studies have reported the presence of Stx-converting phages among UTI isolates, mainly in humans but also in animals (Beutin et al., 1994; de Brito et al., 1999; Mariani-Kurkdjian et al., 2014; Toval et al., 2014b; Cointe et al., 2018; Nüesch-Inderbinen et al., 2018; Gati et al., 2019).

However, few reports have shown the occurrence of diarrhea and extraintestinal infection, in the same patient, as being caused by a single *E. coli* strain. Mariani-Kurkdjian et al. (2014) reported the occurrence of an EHEC strain that caused diarrhea, HUS, and bloodstream infection in an adult in France. This hetero-hybrid pathogen harbors two variants of Stx2, the LEE PAI and one virulence plasmid—(pAPEC-like plasmid) carrying the *ompTp, etsC, iss, hlyF, sitA, cvaA, iroN*, and *iucC* virulence genes—which is often present in APEC and NMEC strains. Moreover, the strain fulfills the intrinsic virulence criteria by Johnson et al. (2003) (Table 1) for consideration of ExPEC (Nüesch-Inderbinen et al., 2018), which may explain the capacity of this pathogen to reach the bloodstream and cause severe neurological symptomatology (Mariani-Kurkdjian et al., 2014; Wijnsma et al., 2017). Other studies from Switzerland and France have shown that this hetero-hybrid pathogen, which belongs to serotype O80:H2 and ST301-A, was also isolated from HUS cases followed by bacteremia (confirmed by blood culture positive) (Soysal et al., 2016; Cointe et al., 2018; Nüesch-Inderbinen et al., 2018). This hetero-hybrid pathogen was also reported in human cases of diarrhea and HUS without bacteremia and from calves with diarrhea in Belgium, Switzerland, and the Netherlands (Fierz et al., 2017; Wijnsma et al., 2017; De Rauw et al., 2019). Cointe et al. (2018) also showed the occurrence of this hetero-hybrid pathogen in human and animal subjects in other European countries, such as Germany, Switzerland, Spain, and Slovakia. It is noteworthy that the report of unusual severe neurological conditions was linked with these hybrid strains, even in the absence of bacteremia, and the HUS occurrence is higher than average (about 80%) (Soysal et al., 2016).

Toval et al. (2014b) have shown other types of ExPEC/STEC hybrid strains isolated from humans in Germany. Some of these were also hetero-hybrid pathogenic strains carrying different subtypes of intimin. In addition, the authors demonstrated the Shiga-toxin functionality in bladder cells and that some strains cause UTI and pyelonephritis in animals. These data were corroborated by another study (Gati et al., 2019) and by the occurrence of one case of diarrhea followed by urosepsis and HUS found to have been caused by another ExPEC/STEC hybrid-pathogen isolated in the Netherlands (Ang et al., 2016).

### ExPEC/EPEC

Diarrhea followed by bacteremia and multiorgan dysfunction was the outcome of the patient from whom this hybrid-pathogen was isolated (Kessler et al., 2015). This hybrid strain called attention also by the fact that a pathogen simultaneously bearing PAIs from DEC and ExPEC could reach additional niches and cause a severe disease. This ExPEC/EPEC strain harbored ExPEC defining virulence factors, LEE, and BFP and belonged to serotype O4:H1 and to ST12-B2, a recognized ST related to extraintestinal infections. The phylogenetic analyses of the strain showed that it clustered with other ExPEC lineages but not with EPEC from phylogroup B2, as the EPEC prototype strain E2348/69 (Kessler et al., 2015). Although not thoroughly characterized, another ExPEC/EPEC hybrid strain of serotype O12:K1: HNM was described, which drove a similar outcome. This strain expressed a functional LEE PAI and invaded diverse cell lineages (Bratoeva et al., 1994).

Not all ExPEC/EPEC hybrids cause a disease in intestinal and in extraintestinal niches. Although rare, these hybrid strains have been isolated from diverse extraintestinal infections from human subjects without diarrhea (Abe et al., 2008; Toval et al., 2014a; Riveros et al., 2017; Valiatti et al., 2019). These strains harbored the LEE, no ExPEC defining virulence factors, and belonged to phylogroups A or B2. However, the reason why these strains caused only extraintestinal infections is unclear.

## Combinations of Virulence Factors With Uncertain Involvement in More Severe Diseases

The studies on the majority of the hybrid/hetero-pathogenic strains that were reported so far lacked information regarding host symptomatology or the expression of virulence factors. Therefore, there is not enough data to determine if the acquisition of new virulence factors necessarily implicates different or more severe symptoms. The combination of virulence traits might not implicate new pathogenicity features or increased virulence because some of the hybrid/hetero-pathogenic strains identified by molecular methods do not express both traits.

### STEC/ETEC

Many reports on STEC/ETEC have shown its high frequency among strains isolated from post-weaning diarrhea and edema disease in piglets (Cheng et al., 2006; Barth et al., 2007, 2011; Beutin et al., 2008; Byun et al., 2013). These hetero-pathogenic strains were also isolated from other animals, food, diarrheic human subjects, and some cases of HUS (Monday et al., 2006; Müller et al., 2007; Barth et al., 2011; Prager et al., 2011; Steyert et al., 2012; Nyholm et al., 2015b; Leonard et al., 2016; Michelacci et al., 2018; Bai et al., 2019; Yang et al., 2020).

Although often reported worldwide, studies conducted in Finland and Sweden, which evaluated a large number of human isolates, demonstrated that the occurrence of STEC/ETEC hetero-pathogens was low (Nyholm et al., 2015b; Bai et al., 2019). In one of these studies, four STEC/ETEC strains were reported, corresponding to 2.05% of the total 195 clinical strains initially characterized as STEC isolated over 15 years. Severe symptomatology promoted by this hetero-pathogen was reported only in piglets, and little is known about the impact of these strains, leading to different clinical conditions in human subjects.

The occurrence of multidrug-resistant (MDR) hetero-pathogenic *E. coli* strains emphasizes their genome plasticity and points to the role of horizontal gene exchange in favoring the emergence of higher virulent MDR clones (Rasko et al., 2011), such as certain STEC/ETEC strains. García et al. (2018) showed a STEC/ETEC strain isolated from pig suffering from post-weaning diarrhea, which carried a plasmid containing multiple resistance genes and another one containing multiple virulence genes. Other authors also reported a multidrug-resistant phenotype among strains of this hetero-pathogenic pathotype (Brilhante et al., 2019).

It is interesting to note the diversity of sequence types (STs), phylogroups, and serotypes (more than 40) that was observed among the STEC/ETEC hetero-pathogenic strains (Table 2). Such diversity suggests that both the ETEC virulence plasmid and Shiga-toxin-converting bacteriophages could be spread to a broad range of genetic backgrounds, including those serotypes related to more pathogenic strains previously described to cause human disease such as O2:H27, O15:H16, O101:H-, O128:H8, and O141:H8 (Nyholm et al., 2015b).

**Table 2:** Summarized characteristics of hybrid- and hetero-pathogens.

	**Isolation source**	**Virulence traits**		**Expression of the virulence traits**	**Disease**	**Outbreaks**	**Serogroup or serotype**	**MLST-phylogroup^a^**	**References**
EAEC/STEC	Intestinal infections	Stx and aggregative fimbriae with *aggR* regulator		Yes	Diarrhea, bloody diarrhea, and HUS	Yes	O104:H4 O59:H- Orough:H- O111:H2 O89:H- O23:H28	ST678-B1 ST26-B1 ST1136-B1	Morabito et al., 1998; Iyoda et al., 2000; Frank et al., 2011; Prager et al., 2014; Lang et al., 2018
EPEC/STEC	Intestinal infections in humans, animals' gut, environment, and food	LEE PAI and Stx	Intimin (various subtypes) and Stx	Yes	Diarrhea, bloody diarrhea, and HUS	Yes	O157:H7 O145 O103 O26 O111	ST11-E ST32cplx-D ST20cplx-B1 ST29cplx-B1 ST29cplx-B1	Eichhorn et al., 2015
	Normal fecal sample	LEE PAI, Stx-2f, and BFP	Identified only in birds	Yes		No	O137:H6	ST2678-B2	Gioia-Di Chiacchio et al., 2018
EPEC/ETEC	Intestinal infections	LEE PAI and LT or ST	Regardless of the presence of BFP	Yes	Watery diarrhea	No	Unknown	ST278cplx-B1 ST1788-A	Dutta et al., 2015; Askari Badouei et al., 2016; Hazen et al., 2017
ExPEC/STEC and ExPEC/EHEC^b^	Diarrhea and extraintestinal infections simultaneously or extraintestinal infections only	Stx and ExPEC intrinsic virulence factors		Yes—both characteristics are expressed In general, harbor Stx2 variant	UTI, hemorrhagic cystitis, HUS, bacteremia	Yes^c^	O2:H6 O76:H19 Ont:H- O80:H2 O145:H-	ST141-B2 ST675-B1 ST10cplx-A ST165cplx-A ST32cplx-D	Mariani-Kurkdjian et al., 2014; Toval et al., 2014b; Gati et al., 2019
ExPEC/EPEC	Extraintestinal infections only or diarrhea followed by extraintestinal infection	LEE PAI only or LEE PAI and ExPEC intrinsic virulence factors	Regardless of the presence of BFP	LA or LAL pattern was observed in some studies	Cystitis, pyelonephritis, UTI-related bacteremia, and diarrhea with multiple organ dysfunction	No	O71 O78:H- O4:H1 O12:K1:H-	Unknown ST2018-B2 ST12-B2 Unknown	Vieira et al., 2001; Abe et al., 2008; Toval et al., 2014a; Kessler et al., 2015; Riveros et al., 2017; Lindstedt et al., 2018; Valiatti et al., 2019
STEC/ETEC	Intestinal infections in human and animals	Stx and ST toxin	Various Stx2 variants; Stx1 is frequently low	Yes, but some strains express just one toxin	Diarrhea, bloody diarrhea, and HUS	No	Various serogroups (>40 serotypes)	ST10cplx-A ST40cplx-B1 ST325-A ST329-A	Nyholm et al., 2015b; García et al., 2018; Bai et al., 2019
ExPEC/EAEC	Extraintestinal infections	Aggregative fimbriae with *aggR* regulator only or with ExPEC intrinsic virulence factors		Yes^d^	Extraintestinal infections not related to diarrhea	Yes	Various serogroups (O5, O6, O11, O15, O18, O78, etc.)	Various ST10cplx-A is the most frequently reported ST69-D ST131-B2	Abe et al., 2008; Olesen et al., 2012; Toval et al., 2014a; Lara et al., 2017; Riveros et al., 2017
ExPEC/ETEC	Extraintestinal infections	ETEC virulence factors only		ST was detected in human, while LT was detected in pigs	Urinary tract infections	No	Unknown	Unknown	de Brito et al., 1999; Riveros et al., 2017

*LEE PAI, locus of enterocyte effacement pathogenicity island; HUS, hemolytic uremic syndrome; BFP, bundle-forming pilus; Stx, Shiga toxin; ST, heat-stable toxin of ETEC; LT, heat-labile toxin of ETEC; UTI, urinary tract infection; LA, localized adherence; LAL, localized adherence-like*.

*ExPEC intrinsic virulence factors—presence of two virulence factors or genetic markers among the following: P fimbriae (pap), S fimbriae (sfa/foc), afimbrial adhesin family (afa/dra), aerobactin (iut/iuc), and capsule from capsular group II (kpsMTII)*.

a *Multilocus sequence typing (MLST) and phylogroup relationship were described as available in Escherichia/Shigella EnteroBase of Warwick Medical School (Zhou et al., 2020); STn°–sequence type number; STn°cplx—sequence type complex; ST complex referred to STs that are grouped into clonal complexes by their similarity to a central allelic profile*.

b *EHEC is used to refer to strains that harbor LEE and Stx simultaneously*.

c *Only EHEC/ExPEC strains were related to an outbreak*.

d *An aggregative adherence pattern was classified in HeLa or HEp-2 cell lineages. The expression of extraintestinal virulence (capacity to cause UTI or sepsis) was observed for some strains in specific animal models*.

### ExPEC Harboring EAEC Virulence Markers

In 1991, a community-acquired UTI outbreak occurred in Copenhagen (Olesen et al., 1994). The genotypic and the phenotypic characterization of the outbreak-related strains showed that they belonged to the O78:H10 serotype, commonly associated with diarrhea. The outbreak strains harbored the complete set of EAEC defining virulence genes, expressed the aggregative pattern of adherence, and belonged to ST10, like the EAEC strains isolated from diarrhea. Interestingly, they were also lethal in an animal model and hence considered as ExPEC, clearly different from other EAEC strains isolated from diarrhea (Olesen et al., 2012). To our knowledge, these findings were the first evidence that some EAEC strains could cause an extraintestinal infection. Nevertheless, it is important to consider that, in the Copenhagen outbreak, there was no evidence that the EAEC strains could cause diarrhea, although diarrheagenic EAEC harboring ExPEC intrinsic virulence factors have already been reported (Nunes et al., 2017).

It is important to note that strains classified as EAEC by their virulence markers are not always causing diarrhea in their hosts. In fact, Nataro et al. (1995) demonstrated that only strain 042 among four EAEC strains studied induced diarrhea in human volunteers, although all the strains carried the EAEC virulence plasmid. Subsequently, many reports came out, registering the isolation of strains from various extraintestinal infections, which presented the EAEC defining genotype and expressed the aggregative adherence pattern *in vitro* (Abe et al., 2008; Nazemi et al., 2011; Toval et al., 2014a; Lara et al., 2017; Riveros et al., 2017; Freire et al., 2020). However, differently from the EAEC strains of the Copenhagen outbreak, the presence of the ExPEC markers are rare among the reported EAEC isolates from extraintestinal infections (Abe et al., 2008; Toval et al., 2014a). Moreover, it is still unclear if the same strain can cause infections in intestinal and in extraintestinal niches.

## Favorable Background for the Mix-up of Virulence Factors

The *E. coli* population can be divided into eight different phylogroups (A, B1, B2, C, D, E, F, and G) (Clermont et al., 2019). Although pathogenic *E. coli* are distributed among all, some pathotypes are frequently assigned to specific phylogroups; for example, ExPEC strains are often referred to as belonging to phylogroups B2 and D, while many DEC strains are of phylogroup B1. In this context, phylogroup A strains are classically related to those that make up the gut microbiota and are avirulent, such as the *E. coli* K-12 prototype strain MG1655 (Leimbach et al., 2013; Clermont et al., 2017; Johnson and Russo, 2018). However, virulent strains, including those associated with both ExPEC and DEC outbreaks, are also assigned into phylogroup A.

Interestingly, some clonal groups inside phylogroup A are frequently reported to be involved in a myriad of infections and multidrug-resistance, such as ST10 (Olesen et al., 2012; Hauser et al., 2013; Riley, 2014; Toval et al., 2014a; Nyholm et al., 2015a; Arais et al., 2018; García et al., 2018; Yamaji et al., 2018). The strains belonging to this ST seem to be very flexible and receptive, bearing many hybrid- and hetero-pathogenic strains (Table 2). These findings suggest that some specific genetic backgrounds could be more permissive to acquire and stably maintain a variety of mobile genetic elements, allowing the emergence of hybrid- or hetero-pathogenic strains.

Gati et al. (2019) have shown that the ST141-B2, which includes both STEC and ExPEC strains, was the origin of some STEC/ExPEC hybrid strains. Accordingly, their analysis suggested that ST141, allocated between well-defined pathogenic clusters, could be a hotspot for the emergence of hybrid strains. These authors have also concluded that the development of the STEC/ExPEC hybrid was a recent event. These events might happen in all clonal groups that harbor more than one of the *E. coli* pathotypes and could not be seen as restricted to any ST or phylogroup. The characteristics of the hybrid/hetero-pathogenic strains are detailed in Table 2.

## What Might be the Consequences of *E. coli* Genome Plasticity?

Some hybrid- and hetero-pathogenic strains have been isolated from human and animal infections since the extensive study of pathogenic *E. coli* strains began. Some of these pathogens were reported long ago, like EPEC/STEC strains, while others, like ExPEC/STEC strains, emerged in recent years and are being pointed only now as the cause of diseases. Although the hybrid- and hetero-pathogenic strains might have appeared long ago, the interest in their significance as more virulent pathogens is a recent phenomenon.

Sequencing technologies have helped to understand the events involved in hybrid/hetero-pathogen evolution, the most common being the transference of virulence genes by mobile plasmids and the acquisition of converting phages. Most of the hybrid strains described until now are related to the STEC pathotype, probably because of the broad host range of Shiga-toxin-converting bacteriophages, since their occurrence has been reported in different species including *Citrobacter freundii* and *Enterobacter cloacae*, which were associated with HUS and one outbreak (Tschäpe et al., 1995; Paton and Paton, 1996). Additionally, Fogolari et al. (2018) recently reported that the presence of the Stx-converting phage could be found in other *Shigella* species besides *Shigella dysenteriae* type 1, mainly in *Shigella flexneri*. Moreover, viable Stx-converting phages were shown to be present in water and sewage (Muniesa and Jofre, 1998; Beutin et al., 2008). Therefore, the prevalence in the environment and the broad host range explain the capacity of these phages to reach new bacteria. Interestingly, there has been no report on the occurrence of the EIEC virulence plasmid among other DEC or ExPEC pathotypes. Nevertheless, the presence in EIEC of some ExPEC virulence genetic markers, including those of UPEC pathogenicity islands, has already been reported (da Silva et al., 2017). However, the reported strains cannot be considered as hybrid pathogens since they do not fulfill the molecular criteria proposed by Johnson et al. (2003) for the classification of intrinsic virulent ExPEC (da Silva et al., 2017).

Currently, there is not enough published data to confirm if hybrid/hetero-pathogens are always more virulent than their parental pathotypes. Although some studies pointed out that the disease prognoses were worst (Bratoeva et al., 1994; Navarro-Garcia, 2014; Kessler et al., 2015; Ang et al., 2016; Soysal et al., 2016; Wijnsma et al., 2017), this question has not been adequately addressed in all hybrid/hetero-pathogenic strains, and more information about the host symptomatology are necessary to better understand their significance.

It is well-known that the *E. coli* genome is a dynamic entity; thus, hybrid- and hetero-pathogens will probably continue to emerge and expand the current set of recognized *E. coli* pathotypes. The great challenge for both human and veterinary medicine will be to promptly identify and hinder these pathogens from spreading and causing massive outbreaks, such as the German outbreak of 2011. Considering that these hybrid/hetero-pathogens can carry virulence-associated makers as well as multidrug resistance genes, there is an urgency to identify them and address appropriate measures of containment.

## Author Contributions

AS and FS designed and conceptualized this review, wrote the first draft, and edited the manuscript. RS and TG wrote and revised it critically. All authors contributed to manuscript revision, read, and approved the submitted version.

## Conflict of Interest

The authors declare that the research was conducted in the absence of any commercial or financial relationships that could be construed as a potential conflict of interest.
